# Analytical Challenges and Strategies for Compound Identification Using Gas Chromatography–Ion Mobility Spectrometry

**DOI:** 10.1021/acs.analchem.5c07242

**Published:** 2026-08-03

**Authors:** Andrés Martín-Gómez, María José Cardador, Laura Pérez-González, Lourdes Arce

**Affiliations:** Department of Analytical Chemistry, Chemical Institute for Energy and the Environment (IQUEMA), 205482 University of Córdoba, Campus of Rabanales, Marie Curie Annex Building, E-14071 Córdoba, Spain

## Abstract

Gas chromatography–ion mobility spectrometry (GC-IMS) is applied for the classification of complex samples using multivariate analysis of volatile organic compounds (VOCs) extracted from the matrix. However, feature identification remains one of its greatest challenges due to feature overlapping, variability of retention index (RI), and limited availability of drift time (DT) values of ions in libraries. Although combined annotation-and-confirmation approaches have been reported in the literature, no systematic and quantitative evaluation of their implementation in GC-IMS has been provided to date. This study addresses that gap by providing practical guidelines and a critical, evidence-based discussion of the challenges and strategies for VOCs identification in GC-IMS. Two approaches were evaluated. First, the use of a commercial library was examined, considering the influence of the employed RI and DT search margins on identification results, and highlighting the limitations of using RI values alone, as well as the decisive role of DT values. Second, the use of analytical standards as a validation tool, demonstrating their essential role in resolving ambiguities caused by feature overlapping and competitive ionization of different VOCs in the ionization chamber, as well as in confirming VOCs not identified with the library due to missing DT values. In addition, a simplified workflow to achieve a reproducible identification of GC-IMS features is provided. A mixture of 45 standards and a virgin olive oil sample were used as model systems, demonstrating that the combined approach with library search and standard analysis yields more reliable identifications.

## Graphical abstract

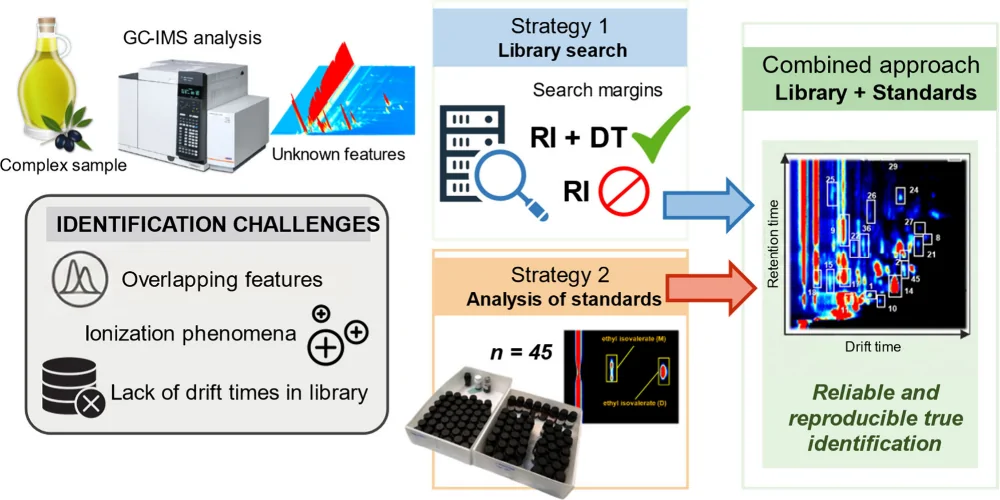

## Introduction

The use of gas chromatography–ion mobility spectrometry (GC-IMS) has expanded rapidly in recent years. A search in the *Web of Science* database of the topic “GC-IMS” filtering to articles and reviews yielded 1733 publications through March 2026. Of these, roughly 91.2% have been published within the last five years (2021–2026). GC-IMS is a highly versatile technique whose main field of application is the classification of samples through their volatile profile, either with targeted or nontargeted methodologies, addressing identification of compounds or not. Its use has extended to many areas and a broad range of matrices,^1^ such as the analysis of agri-food products^2,3^ and clinical diagnosis.^4^ This rapid growth of articles published using GC-IMS has been accompanied by an increasing interest in data processing strategies for nontargeted multivariate approaches. Although these may introduce higher noise into the data, they minimize the risk of discarding relevant information.^5^ In fact, comparisons between targeted and nontargeted models generally have shown better performance for the latter,^6–8^ even though the outcome depends on the diversity and volume of samples analyzed. While less common, there is a growing interest in applying targeted GC-IMS approaches that take advantage of its high sensitivity, enabling the detection of analytes at very low concentration. As an example, targeted approaches have been successfully employed to identify and quantify ethanol to discriminate olive oil categories,^9^ alkylpyrazines to monitor the roasting of hazelnut,^10^ nitrosamines to determine the quality of drinking water,^11^ and ethyl carbamate to ensure the safety of alcoholic beverages.^12^ Beyond its established role in sample classification and fingerprinting, the intrinsically high sensitivity of IMS as a detector constitutes an analytical advantage often underappreciated. The IMS cell operates at atmospheric pressure and relies on soft chemical ionization (primarily proton exchange which, unlike the electron impact (EI) source of a conventional GC–MS, does not require analytes to be present above the concentrations needed to produce a detectable signal in full-scan mode. As a result, GC-IMS can detect volatile organic compounds (VOCs) at the subppb level directly from the sample headspace, without any preconcentration step.

This sensitivity advantage has important consequences for untargeted analysis. When VOCs are screened by HS-GC-MS, the detectable profile is limited to those analytes sufficiently abundant in the headspace. To extend coverage and improve sensitivity, HS-GC-MS is often coupled with preconcentration techniques such as SPME, which also introduces selectivity biases, depending on the fiber polarity. A medium-polar CAR-PDMS fiber, for instance, will preferentially retain organosulfur compounds and low-molecular-weight polar analytes, whereas other chemical classes may be poorly recovered even after optimization of extraction conditions. Changing fiber chemistry or exposure conditions will reveal a different subset of the volatile fraction, but no single fiber provides exhaustive coverage. Thus, the VOCs that remain invisible to a given analytical setup cannot be targeted for optimized extraction, simply because the analyst does not know they were present in the sample.

This analytical blind spot has been demonstrated in a comparative study by our group,^13^ where the HS-GC-IMS analysis of cultures of *Pseudomonas* detected three VOCs (2-methylbutan-1-ol, pentan-2-one, and 4-methylpentan-2-one) that were absent from both HS-GC-MS chromatograms with or without SPME coupling. Injection of the respective analytical standards confirmed that all three compounds were genuinely present in the headspace at concentrations between 2 and 4 times below the detection limits of HS-GC-MS. This finding implies that, had the analysis been performed exclusively by GC-MS, these three metabolites would have gone entirely undetected, and no targeted preconcentration strategy could have been designed for them. Similar results have been reported in food matrices using HS-GC-IMS and HS-GC-MS instruments operated under identical headspace conditions for the characterization of Iberian dry-cured ham VOCs:^14^ 16 out of the 25 compounds identified in the samples were detected exclusively by HS-GC-IMS. Similarly, in the monitoring of fresh pork spoilage, butanal and ethyl acetate (two of the VOCs most strongly correlated with microbial growth) were detected only by HS-GC-IMS.^15^ These observations are consistent with published LOQ data that confirmed a sensitivity advantage of GC-IMS, with respect to GC-MS, up to 26-fold for common olive oil, depending on the compound.^16^ Therefore, before a targeted HS-SPME-GC-MS method can be designed and validated for a given matrix, a nontargeted screening step with GC-IMS is valuable to establish which compounds might be present, particularly at trace levels.

Despite its advantages, GC-IMS faces certain limitations when identifying VOCs in chemically rich matrices. These include low selectivity resulting from a broad ionization and reactant ion peak (RIP) saturation at high analyte concentrations.^5^ Thus, the main advantage of IMS compared to mass spectrometry (MS)—its higher sensitivity—might become a drawback when the aim is the identification of nontrace compounds. These factors constitute a bottleneck that limits the incorporation of the technique in targeted qualitative studies. Complex samples typically generate large numbers of features in GC-IMS plots, thus accurate identification requires careful consideration of their retention index (RI) and drift time (DT). Likewise, these are directly influenced by the chromatographic column, physicochemical properties of the analytes (e.g., volatility and proton affinity, PA), and ionization phenomena.

Published studies that have attempted feature identification with GC-IMS either do so by comparing the RI and DT of sample features with (a) those obtained from analyzed standards, or (b) those present in commercial libraries. The first strategy has proven useful for achieving the true identification of a limited number of VOCs for specific applications, such as the detection of defective dry-cured Iberian ham,^14^ the differentiation of microorganisms contributing to cheese aroma,^17^ or the characterization of bacterial emissions.^13^ However, the preparation and analysis of standards is a time-consuming and costly process, and the scope of this approach is constrained by the availability of reference compounds. The second strategy, based on the comparison of unknown features with data stored in libraries, greatly facilitates the process of annotation. However, current databases integrate far fewer DT values than RI values (compiled from NIST). Thus, while libraries provide a useful tool for the rapid putative identification of features in topographic plots, they are limited.

In general terms, the most cited (>100) studies published from 2020 onward that have tried to identify GC-IMS features have relied exclusively on software libraries for this purpose, whereas previously the analysis of standards was more common; so far as to use, e.g., 42 ^18^ or 31 ^19^ standards to achieve a true identification of plot features. Table 1, which includes identification or annotation results found in papers with the topic “GC-IMS” and >100 citations, shows this change in trend. Still, authors usually fail to specify the confidence levels and criteria applied, or whether the proposed correspondences were validated or not with analytical standards. Notice in Table 1 that using only a library several authors “identified” more than 30 VOCs, but they did not confirm using standards, so they only achieved a putative identification at best. Moreover, occasionally the terms “feature” or “signal” (which correspond to individual ions) and “VOC” (which corresponds to the neutral compound before it generates one or more characteristic ions) were confused. As an example, in a published paper^20^ “a total of 71 compounds were identified through GC-IMS analysis, of which 52 were classified as monomers or dimers and 19 remained uncharacterized”. In another,^21^ “49 flavor substance (sic) were identified”. In both of them, the authors were referring to features of individual ions, not compounds. In these cases, it may appear that the authors have annotated—standards were not used—more VOCs than they actually have.

**Table 1:** Overview of GC-IMS Feature Identification in Studies with “GC-IMS” as a Topic and >100 Citations Published between 2015-2026 According to the *Web of Science* Database, Consulted in March 2026^a^

Reference	Year	Sample matrix	Located features	Identified features	Identified VOCs	Identification method
^22^	2015	Olive oil	–	–	26	B
^23^	2017	Olive oil	–	7	4	B
^24^	2017	Essential oils	–	115	50	B + C
^6^	2018	Iberian ham	44	5	3	A + B
^25^	2018	Honey	–	23	18	B
^26^	2019	Eggs	35	–	5	B + C
^19^	2019	Olive oil	–	35	24	B
^27^	2019	Fish	–	41	25	A
^28^	2019	Edible fungus	–	43	25	A
^29^	2019	Honey	141	–	8	A^b^ + C
^30^	2020	Sorghum	–	–	43	A
^31^	2020	Peppers	–	54	45	A
^32^	2020	Jinhua ham	–	37	25	A
^33^	2020	Human breath	80	10	8	–
^34^	2020	Dry-cured pork loin	–	54	48	A
^35^	2020	Tofu	–	40	24	A
^36^	2020	Dry-cured fish	–	30	22	A
^37^	2021	Tilapia	40	28	20	A
^38^	2021	Soybeans	80	50	42	A
^39^	2021	Sichuan pepper	84	62	49	A
^40^	2021	Shiitake	–	69	55	A
^41^	2021	Dry-cured ham	–	63	45	A
^42^	2021	Lamb meat	–	66	41	A
^43^	2022	Tea	–	51	47	A
^44^	2022	Yak meat	64	45	34	A
^45^	2022	Duck meat	50	35	28	A
^46^	2022	Surimi	–	59	41	A
^47^	2022	Tea	60	45	33	A^c^
 ^48^	2022	Braised chicken	–	37	31	A + B
^49^	2023	Shrimp paste	63	31	21	A
^50^	2023	Tea	–	78	63	A
^51^	2023	Tea	–	94	72	A

a Legend: A, Library; B, GC-IMS analysis of standards; C, Correlation of experimental RIs with GC-MS data. −: Not included in the paper.

b Includes information about the library search margin used.

c The library was enriched with in-house data.

Alternative approaches to improve compound identification in GC-IMS involve the coupling of GC to MS and IMS in parallel, which provides an orthogonal dimension of information based on molecular mass and fragmentation patterns. The potential for detection of VOCs in complex matrices using GC-IMS is not comparable to that of GC-MS; IMS detectors are not able to fragment analytes. The combination of RIs (GC), DT (IMS), and mass spectral data (MS) can substantially increase confidence in compound assignment. A possible strategy consists of performing GC-IMS/MS analyses using two chromatographic columns with the same characteristics—separately—and correlating both datasets through RIs.^52^ However, this is a suboptimal strategy, because this approach may introduce uncertainty due to RI shifts, even if a single column type is used. In contrast, parallel configurations in which GC is coupled simultaneously to IMS and MS using a single chromatographic column^53–55^ provide a more robust approach, as all detectors analyze the same eluting compounds under identical conditions. This enables a truly orthogonal confirmation, reducing ambiguities associated with coelution and improving the reliability of compound identification. Despite these advantages, such coupled systems remain technically demanding due to the different operating conditions required by IMS (atmospheric pressure) and MS (vacuum), as well as their higher cost and complexity compared to the use of a single detector coupled to a GC, which may limit their accessibility for routine applications.

Therefore, in view of the notable rise in targeted GC-IMS applications and the presence of methodological ambiguities, there is a clear need for a systematic evaluation of current feature—and compound—identification practices. The main objective of this work is to establish evidence-based guidelines to identify with GC-IMS. Specifically, this study (a) examines the process of annotation using the currently available library of standards, and (b) evaluates the role of RI and DT values of analytical standards on true identification of GC-IMS features. Using virgin olive oil (VOO) as a real sample and a mixture of 45 standards, we aim to provide a framework for a more-reliable identification in GC-IMS.

## Materials and Methods

### Reagents and Standards

A total of 45 analytical standards including 10 aldehydes ((E)-2-pentenal, (E,E)-2,4-hexadienal, 2-methylpropanal, benzaldehyde, butanal, heptanal, hexanal, nonanal, octanal, and pentanal); 12 ketones (1-octen-3-one, butan-2-one, pentan-2-one, hexan-2-one, heptan-2-one, octan-2-one, nonan-2-one, 4-methylpentan-2-one, 6-methyl-5-hepten-2-one, cyclohexanone, octan-3-one, and diacetyl); 12 alcohols (octan-1-ol, 1-octen-3-ol, pentan-1-ol, 1-penten-3-ol, heptan-2-ol, 2-methyl-3-buten-2-ol, 2-methylbutan-1-ol, 3,3-dimethylbutan-1-ol, 3-methylbutan-1-ol, *cis*-2-penten-1-ol, octan-3-ol, and hexan-1-ol); 7 esters (ethyl acetate, ethyl isovalerate, ethyl propanoate, ethyl hexanoate, heptyl ethanoate heptyl-acetate, propyl butyrate, and methyl butanoate) and other compounds, including a carboxylic acid (acetic acid), two heteroaromatic compounds (2-methylpyrazine and 2,5-dimethylpyrazine), and a terpene (R-limonene) were analyzed by GC-IMS. All standards were commercially supplied by Sigma–Aldrich (Madrid, Spain). The selection was based on their presence in various agri-food matrices that have been analyzed over the years in our research group, such as olive oil and Iberian dry-cured ham.

Stock solutions (1000 mg/L) of each standard were prepared by dissolving appropriate amounts in Milli-Q ultrapure water (Millipore, Bedford, MA, USA) and later stored at 4 °C. Likewise, intermediate (100 mg/L) and working (1 mg/L) solutions were freshly prepared daily by dilution. Individual standards and standard mixtures used for identification were prepared and analyzed at a concentration of 1 mg/L. For each measurement, 1 mL of the working solution was placed into 20 mL headspace vials (Labbox Labware, S.L., Barcelona, Spain). In addition, 1 g of a sample of virgin olive oil (VOO) supplied by Sovena España S.A. (Brenes, Spain) was placed into a 20 mL headspace vial and analyzed.

A quality control mixture was prepared daily by dissolving six high-purity (≥99%) ketones (butan-2-one, pentan-2-one, hexan-2-one, heptan-2-one, octan-2-one, and nonan-2-one) at 0.5 mg/L in ultrapure water (18.2 MΩ cm^–1^, Milli-Q Plus system, Millipore, Bedford, MA, USA). This mixture was analyzed prior to each vial batch, as a quality control for subsequent analyses.

### Instrumentation

Analyses in GC-IMS were performed by using an integrated Agilent 7697A headspace sampler connected by a transfer line to an Agilent 8860 gas chromatograph (Agilent, Santa Clara, CA, USA) and a standalone ion mobility spectrometer (G.A.S. Gesellschaft für Analytische Sensorsysteme mbH, Dortmund, Germany) with a 9.8 cm drift tube and a ^3^H ionization source. Samples were incubated for 15 min at 60 °C. Then the vial was pressurized to 14 psi for 1.5 min and a 1 mL of the headspace volume was injected in split mode 1:5 during 30 s with a split flow of 5 mL/min. The sampling loop and transfer line were maintained at 100 and 110 °C, respectively. GC separation was carried out using a nonpolar 30 m HP-5 column (Agilent Technologies, Santa Clara, CA, USA) with stationary phase composition of 5%-phenyl-methylpolysiloxane with 0.32 mm of internal diameter and 0.5 μm of film thickness. Helium (99.999% purity, Air Liquide, Madrid, Spain) was used as a carrier gas with a constant flow rate of 1.0 mL/min. The oven temperature program was as follows: initial temperature of 40 °C held for 3 min, ramped to 100 °C at a rate of 5 °C/min, then increased to 130 °C at 15 °C/min. The final temperature (130 °C) was maintained until the end of the analysis, resulting in a total run time of 27 min.

After separation in the GC column, VOCs entered the ionization chamber of the IMS module (G.A.S. Gesellschaft für analytische Sensorsysteme mbH, Dortmund, Germany), whose detector was working at positive polarity. Nitrogen (purity of ≥99.999%) (Air Liquide España, S.A., Madrid, Spain) was used as drift gas at a flow of 150 mL/min. The drift tube temperature was maintained at 45 °C with an injection pulse width of 150 μs. Signal averaging was performed every 32 spectra with a repetition rate of 30 ms. The applied voltages for drift, blocking, and injection were 237, 40, and 2500 V, respectively.

### Software and Data Analysis

VOCal 0.2.9 software (G.A.S. Gesellschaft für Analytische Sensorsysteme mbH, Dortmund, Germany) was employed for data visualization and compound annotation. The GC-IMS data are visualized as two-dimensional topographic plots, where RT (s) or RI is displayed on the *y*-axis, DT (ms or dimensionless if it is RIP relative) on the *x*-axis and feature intensity (V) is represented by a color scale. Annotation was performed by matching detected features with the integrated VOCal library, which incorporates RI information from the NIST2020 database and the G.A.S. IMS drift time database, thereby enabling the assignment of features to known VOCs. RIs were calculated by normalizing with the experimental RTs of 9 *n*-ketones (C4–C9), used as external standards.

## Results and Discussion

### Key Analytical Considerations Prior to VOCs Identification with GC-IMS

Before proceeding with the identification of features in GC-IMS plots, several analytical aspects must be considered to ensure reliable and reproducible results. First and foremost, we need to clearly define the term “identification”, as different terms are used to represent distinct levels of confidence about a molecule’s structure. As an example, the Metabolomics Standards Initiative established a four-level hierarchy to standardize metabolite identification with high-resolution mass spectrometry (HRMS):^56,57^ (1) truly identified; (2) putatively identified or annotated (e.g., without chemical reference standards, based upon physicochemical properties and/or spectral similarity with public/commercial spectral libraries); (3) characterized class (e.g., based upon characteristic physicochemical properties or by spectral similarity to known compounds of a class); and (4) unknown—although these metabolites can still be differentiated. In addition, Schymanski et al. proposed an alternative list of confidence levels for HRMS:^58^ (1) confirmation through the reference standard, which is analyzed and matches the compound retention time and mass spectrum; (2) tentative identification if the spectrum matches the library (level 2a) or the fragmentation pattern makes sense for that structure (level 2b); (3) class identification (e.g., “aldehyde”) with a list of several candidates; (4) molecular formula without any specific structure; and (5) exact mass of interest. Therefore, in order to achieve true identification, the analysis of a reference standard is required, whose retention time and mass spectrum match the signal of interest. These criteria might be partially translated to GC-IMS as described in Table 2, assuming that the fragmentation (*m*/*z*) achieved in HRMS is comparable to the additional orthogonal dimension represented by the DT; IMS instruments with tritium source rely on soft chemical ionization, which rarely fragments.

**Table 2:** Comparison of Confidence Levels for Compound Identification between HRMS and GC-IMS Techniques

Confidence level	HRMS	GC-IMS equivalent
Level 1 (confirmation)	Match with reference standard	Match with RI and DT (RIP relative) of all the features of the reference standard
Level 2 (putative identification)	Match with spectral libraries (e.g., NIST)	Match with both RI (NIST) and DT (RIP relative) of library. It is harder than with MS, as IMS libraries are smaller and less universal
Level 3 (class)	Based on fragmentation patterns showing chemical family traits	A similar but not equal level can be achieved if the features of its chemical family are peculiar (e.g., “dimer tailing” in alcohols) but these cases are rare and it is less specific than MS fragmentation
Level 4 (molecular formula)	MS provides the molecular formula	Impossible, as GC-IMS cannot count atoms. It can only determine if the molecule has a specific shape/size taking into account the *K*_0_ or CCS^a^
Level 5 (exact mass/feature position)	MS determines the exact mass (*m*/*z*) of interest	Impossible, as GC-IMS does not determine mass. It would be equivalent to an “unknown feature”

a *K*_0_ is the reduced ion mobility constant, CCS is the collision cross-section.

According to Table 2, confidence level 5 in both GC-HRMS and GC-IMS allows a feature to be traced in future studies. In the case of GC-IMS, this only happens if its RI and DT are defined. Nevertheless, DT is dependent on, e.g., pressure and electric field magnitude and it is not as useful for interlaboratory studies. In the IMS drift tube, if the temperature rises or the pressure drops, the gas becomes less dense and the ions fly faster. Ion mobility (*K*) calculated as *K* = *v*_d_/*E* (where *v*_d_ represents the ion drift velocity and *E* the strength of the electric field) and its normalization (*K*_0_) to standard temperature and pressure (273.1 K and 101.3 kPa, respectively) removes the dependence on instrumental parameters and ambient conditions.

On the other hand, confidence levels 4 and 3 could be hardly achieved with GC-IMS, since it cannot determine molecular formulas, and feature traits related with specific VOC classes are scarce, respectively. Therefore, without a library match, we fall straight from level 2 (putative identification) to level 5 (unknown feature); thus, we miss the middle ground of uncovering the formula without a structure. In contrast, GC-HRMS can infer the exact atoms in the molecule (e.g., C_10_H_16_O) and provides clues while the structure is still unknown. For level 2 identification, the RI for GC-HRMS is considered just “desirable additional evidence”, while, for GC-IMS, a RI match (calculated using *n*-ketones, as previously described) is mandatory but insufficient by itself; it must always be accompanied by a DT match, as it will be demonstrated in the following section. In any case, level 1 identification with GC-IMS, obtained through standard analysis, would still be far from the confidence that can be obtained with a multiple fragmentation obtained with MS/MS or IMS-MS hyphenation.

Second, there are inherent aspects of IMS that may present analytical challenges for compound identification. Ionization in IMS with tritium sources typically occurs via proton association reaction. In positive polarity mode, reactant H^+^(H_2_O)_*n*_ ions associate to neutral analyte molecules with higher PA.^59^ Thus, compounds with a PA lower than water (699 kJ/mol) such as alkanes are not easily ionized with tritium sources^18^ and therefore will not be detected, while other authors have done so using (Ni-63)^60,61^ or corona discharge^62^ sources. Consequently, the PA of the analytes must be carefully considered when interpreting GC-IMS data.

Another important aspect to consider is the matrix effect and the low volatility of certain compounds when using a headspace (HS) sampler coupled with GC-IMS. If they have high affinity for the matrix or high boiling points, they will require heating the sample at elevated temperatures to promote their release. In aqueous matrices, where heating near or over 100 °C may introduce moisture into the IMS ionization chamber, this would be detrimental to ionization efficiency and cause unstable feature intensities and DTs.^63^

It is also important to consider the effect of peak tailing, a distortion that results in elongated features. This phenomenon is evident in terpenes and other high-boiling VOCs.^64^ The resulting overlapping of features may hamper their identification as some will be completely masked by the tail of others. Recent studies point to condensation inside the IMS cell as one of the main causes of peak tailing, which is fixed by increasing the drift tube temperature, thus improving peak symmetry.^65,66^ Feature overlapping represents a critical challenge in the identification of VOCs^67^ in complex samples where hundreds of features coexist, many of them with similar RI and DT values. Thus, without adequate resolution, overlapping may lead to the misidentification of features. Fortunately, advanced deconvolution methods, such as watershed segmentation,^68^ are promising tools to fix this problem. Another mitigating factor is the IMS ability to generate multiple ionic species from a single VOC, (e.g., proton-bound monomers, dimers, and trimers), which can be helpful in cases of partial overlapping. The concurrent presence of different species enhances the confidence when identifying a VOC, as the observation of their features serves as mutual confirmation. If one of the features is concealed, other may still be visible, thus giving away the identity of the original VOC. On the other hand, and as a general rule, when the analyte concentration is close to its limit of detection, only the protonated monomer is observed. Dimer is generated progressively when the concentration of the VOC increases, at the expense of the monomer. As usual, there are also exceptions to this trend: For instance, the protonated monomer of 6-methyl-5-hepten-2-one does not dimerize even at concentrations as high as 4 mg/L,^18^ which may be attributed to steric effects hindering the stabilization of the dimer, although the precise mechanism remains unresolved.

An additional consideration concerns the relative position of ion features with respect to the RIP. The product ions of the majority of VOCs appear after the RIP, as they generally have a larger collision cross-section (CCS) and, consequently, lower mobility. Nonetheless, features corresponding to ions with higher mobility than the RIP may appear on the topographic plot. This phenomenon has been described in the present study (see Figure S1) and/or by other authors for 2-methyl-3-buten-2-ol, 1-penten-3-ol,^45^ and *cis*-2-penten-1-ol.^18,69^ While these alcohols share double bonds in their structure, this behavior has not been observed in *trans*-2-hexen-1-ol, whose features appear after the RIP.^18^ Moreover, mobilities higher than the RIP have been also observed in ion features of dimethyl sulfide,^13,27^ acetone,^70^ (H_2_O)_*n*_NH_4_^+^, and (H_2_O)_*n*_NO^+^.^71^ Despite these reports, this phenomenon remains scarcely discussed in literature and no mechanistic explanation has been yet proposed. Therefore, understanding this behavior is essential to avoid confusing features with noise or artifacts.

Adding to this complexity, product ions of one compound may interact with those of another, leading to the appearance of additional features in the topographic plot. This phenomenon has been previously observed in a mix of ethyl isovalerate and *trans*-hexen-2-al.^18^ While each compound individually produced two features (monomer and dimer), their mixture generated byproduct ions, thereby complicating the identification of their features in complex samples.

### Identification of VOCs Using Library

Before describing the annotation results obtained with the library, it is important to emphasize that the quantitative framework presented here, such as the relationship between search window size and annotation probability, was inferred using VOCal software integrated with the G.A.S. IMS platform. However, the conceptual approach is broadly applicable, as any library search tool operating on RI and DT values will exhibit an analogous tradeoff between sensitivity (risk of false negatives with narrow windows) and specificity (risk of false positives with wide windows). The numerical thresholds proposed below should be understood as validated starting points for the specific combination of software, database, and instrument used here, and may require adjustments for other systems. At the time of carrying out this work, the authors have only found a single commercial database that allows separated compounds to be identified with GC-IMS.

The identification of VOCs in GC-IMS is challenging due to factors discussed in the previous section. The most current library allows the putative identification of unknown features in the GC-IMS plot through comparison of (a) their experimental RI with the RI records available in the NIST database and (b) their experimental DT with the DT records of standards, included as in-house data. Despite the scarcity of DT data in the library (just from ∼200 VOCs vs RIs from ∼83 000 VOCs), the software allows operators to upgrade databases with additional experimental data obtained after analyzing standards, thereby extending its capability for identification. In this section, the potential of libraries for identifying VOCs in complex samples was evaluated. While identification achieved with this method is just putative, it is the only one used by most of the authors who identify GC-IMS features, as previously discussed. In addition, only a minority of them clearly explain the procedure they have followed to reach their conclusions. Therefore, it is difficult to verify whether there have been any misidentified markers in already published papers.

The identification process with a library begins with a normalization of RT using a homologous series of C4–C9 ketones as external standards. Their position in the plot allows the conversion of RT to RI. After normalization, the operator is able to search for candidate VOCs by comparing the experimental RI and DT of unknown features with library records. For this purpose, the user must first define the search margins for RI and DT, which restricts the query to a specific region of the plot (see Figure 1). Then, after the position of a feature is selected, the library will provide a filtered list of candidate VOCs with RI and DT values within that region.

**Figure 1:**
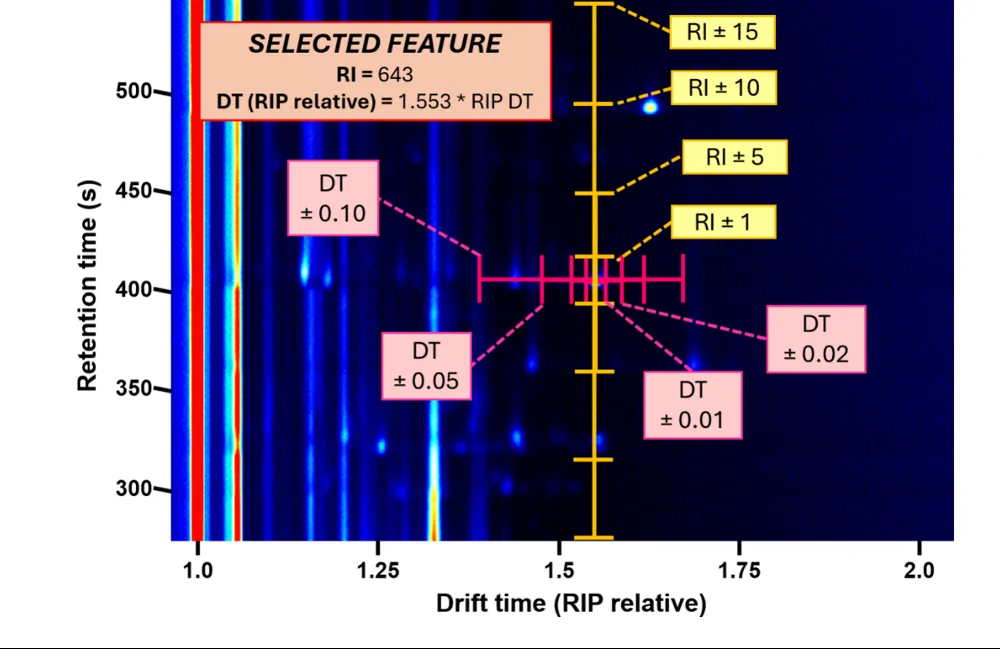
Example of a library search within the GC-IMS plot of a VOO sample that shows a selected feature and a representation of the size of different predefined search margins for DT (pink) and RI (yellow).

The adequate selection of search margins for RI and DT plays a critical role for reliable annotation of GC-IMS features. With the aim of determining their optimal values, the results of library searches of features corresponding to 34 VOCs using different RI margins (±15, ±10, ±5, and ±1) were compared. These 34 VOCs were selected because their features were successfully identified at 1 ppm in the test mixture, which contained 45 analytical standards. For each search margin, the number of candidate VOCs returned by the library within the plot was documented. Then, the probability of correct identification was calculated as the ratio between 1 (the correct match, verified with the analysis of the individual standard) and the total number of matches. As an example (see Table 3), if ethyl isovalerate is searched using its experimental RI of 851.8 with a search margin of ±1, 14 possible matches can be found. Therefore, the probability (%) of finding the only correct match (ethyl isovalerate) between those 14 VOCs is 1/14, which is equivalent to 7.1%. In general terms, applying a RI margin of ±15 yielded many candidate VOCs (≥22) for each feature, thus the chance of matching with the correct one was extremely low (a median of 1.7%). Therefore, such a wide RI margin it is not even useful for screening, let alone for putative identification. On the other hand, the maximum median chance of a correct match (10.0%) was achieved using a ±1 search margin, which is still insufficient for reliable identification. However, it is important to note that when the RI search margin was reduced to an overly strict value (such as ±1); in some cases, it was not possible to find the match of a standard with its feature in the topographic plot. This means that the chance of correctly identifying that feature might be reduced to 0%. This happened for 11 of the 34 studied VOCs using a ±1 margin for RI (see its column in Table 3), which represents 32.3% of the studied VOCs. Also, if no RI values of a VOC are included in the library, the probability of identifying it will be 0% in all cases (e.g., 3,3-dimethylbutan-1-ol in Table 3). Taking these results into account, we can conclude that the use of a narrow RI search margin is recommended; however, based on the data obtained with the VOCal library and the instrumental method used, the margin should not be lower than ±5 RI units in order to avoid false negatives. It must be noted that this threshold was determined for a set of 34 VOCs under the analytical conditions described in the Instrumentation section, and its generalizability to other compounds, matrices, or GC-IMS configurations has not been directly tested. In particular, the RI variability recorded in the NIST database, which is integrated within VOCal library, reflects measurements across a wide range of chromatographic conditions and instruments,^72^ so laboratories working with different column types or temperature programs may observe different optimal margins. Therefore, this value should be treated as an evidence-based recommendation for similar nonpolar columns or configurations rather than a universal constant.

**Table 3:** Probabilities of a Correct Match Identifying Features of 34 VOCs in the GC-IMS Plot Using Different Search Margins of RI

		Probability^a^ (%)			
VOC	Actual RI	Search margin ±15	Search margin ±10	Search margin ±5	Search margin ±1
diacetyl	609.7	4.3	6.2	7.1	0^b^
butan-2-one	617.9	4.3	5.6	8.3	12.5
2-methyl-3-buten-2-ol	631.5	4.5	6.3	8.3	33.3
acetic acid	647.3	3.8	5.6	0^b^	0^b^
1-penten-3-ol	682.2	3.8	5.0	7.7	16.7
pentan-2-one	691.2	3.8	4.5	7.1	20.0
pentanal	700.8	3.6	5.3	7.1	16.7
ethyl propanoate	712.3	3.3	5.3	8.3	16.7
methyl butanoate	721.9	3.4	4.5	7.1	33.3
4-methylpentan-2-one	736.2	3.6	4.3	5.9	16.7
*trans*-penten-2-al	752.5	2.7	3.7	5.0	0^b^
*cis*-2-penten-1-ol	774.3	2.3	3.0	0^b^	0^b^
pentan-1-ol	779.9	2.1	3.1	5.0	16.7
hexan-2-one	792.0	1.8	2.5	4.2	10.0
hexanal	799.2	1.7	2.3	3.4	11.1
3,3-dimethylbutan-1-ol	799.6	0^c^	0^c^	0^c^	0^c^
2-methylpyrazine	824.6	1.9	2.9	5.3	20.0
ethyl isovalerate	851.8	1.5	1.9	2.8	7.1
hexan-1-ol	869.6	1.2	1.7	2.8	5.9
heptan-2-one	889.0	1.7	1.8	3.2	6.7
cyclohexanone	889.3	1.7	2.0	0^b^	0^b^
(E,E)-2,4-hexadienal	908.3	1.5	1.9	3.3	0^b^
2,5-dimethylpyrazine	910.9	1.4	2.0	3.1	10.0
benzaldehyde	965.6	1.3	2.0	2.9	9.1
1-octen-3-one	987.0	1.0	1.3	2.0	0^b^
1-octen-3-ol	989.7	1.0	1.3	1.9	0^b^
6-methyl-5-hepten-2-one	997.4	1.0	1.5	1.8	0^b^
octan-2-one	1003.0	0.9	1.2	1.8	3.8
ethyl hexanoate	1010.2	0.9	1.1	1.9	5.3
R-limonene	1035.9	1.2	1.6	2.7	6.3
octan-1-ol	1083.9	1.0	1.4	2.6	9.1
nonan-2-one	1097.0	0.9	1.2	2.0	0^b^
nonanal	1108.0	0.9	1.2	2.0	5.9
heptyl ethanoate	1115.5	0.9	1.5	2.4	9.1
					
	**Median**	1.7	2.0	3.3	10.0
	**% RSD**	57.1	58.3	53.8	61.2
	*n* with % = 0	1/34	1/34	4/34	11/34

a Probability was calculated as the ratio between 1 (the correct match, verified with the analysis of the individual standard) and the total number of matches provided by the library.

b The library was not able provide a match of the feature with its actual VOC as the values of the RI of said VOC in the library were outside the search margin.

c The library did not contain any RI data of the VOC.

Although all analyses described in this study were performed using G.A.S. VOCal software, the methodological principles underlying the evaluation of search margin effects are applicable to any annotation system that employs windowed searches over RI and DT. In general terms, the library search procedure consists of defining a rectangular query region in the two-dimensional RI–DT space, centered on the coordinates of an unknown feature, and retrieving all database entries whose reference values fall within that region. The probability of a correct match is therefore a function of the density of database entries in that region and the size of the search window. This methodology is independent of the specific software implementation, and the recommended search tolerances of this study (±5 RI units, ±0.02 RIP-relative DT) should be interpreted as evidence-based guidance transferable to analogous commercial or open-access tools. Researchers using alternative platforms, such as MassHunter with IMS plug-in, or custom Python-based pipelines operating on exported peak tables, are encouraged to validate these tolerances for their systems, as instrument-dependent variability in RI and DT measurements may require adjustments.

In view of the poor identification performance of the library using RI alone, it is almost mandatory to resort to DT as an additional parameter. Although the library contains far fewer VOCs with DT than with RI (200 vs 83 000), if DT is available, it greatly increases the likelihood of success, as it represents an additional search axis. To prove this point, we compared the identification success of the feature of pentan-2-one dimer using different RI and DT search margins. As can be seen in Table 4, without DT available the maximum chance of success in identification remains very low (20%) even with the lowest RI search margin (±1). On the other hand, with DT search the number of candidates retrieved by the library was markedly reduced, thus the chance of success increased, reaching 100% from a search margin of 0.02 downward, with a constant ±10 search margin for RI. Nevertheless, we have to take into account that DT matches do not differentiate between ionic species: When a match is found, the library used in this work does not tell if it corresponds to, e.g., a monomer or a dimer. Therefore, it is up to the operator to decide which one it is. This can generally be inferred because dimers have higher drift times than monomers.

**Table 4:** Probabilities^a^ (%) of a Correct Match When Putatively Identifying Features of Pentan-2-one Dimer (Experimental RI = 691.2, Experimental RIP Relative DT = 1.370) in the GC-IMS Plot Using Different Combinations of Search Margins of RI and DT

	DT search margin				
RI search margin	No DT search	±0.10	±0.05	±0.02	±0.01
±1	20%	100%	100%	100%	100%
±5	7.1%	25%	50%	100%	100%
±10	4.5%	16.6%	33%	100%	100%
±15	3.8%	12.5%	25%	50%	50%

a Probability was calculated as the ratio between 1 (the correct match, verified with the analysis of the individual standard) and the total number of matches, provided by the library.

As a conclusion, according to the results of Tables 3 and 4, for feature identification using the GC-IMS library, a search tolerance for DT of ±0.02 or less and a RI tolerance of ±5 (not less) is advised. These values reduce the list of candidates and at the same time prevent false negatives, although the optimal setting should always be tested and validated for each specific application. Overall, our findings show that RI data alone is clearly insufficient for VOC identification, and that DT values are a must for minimally reliable results. It has been demonstrated that the careful definition of search margins is crucial for GC-IMS qualitative analysis and should be documented to guarantee accuracy and reproducibility of results. After a careful revision of GC-IMS papers published between 2020 and 2026, it can be confirmed that no author in that period has indicated which margins were employed in the library search. Still, the information provided by the library is only useful for an initial screening of VOCs. The results obtained should be verified afterward through the injection of individual analytical standards.

### Improving VOC Identification in GC-IMS: The Role of Standards

As described in the previous section, the GC-IMS VOC library provides support for putative feature identification. However, its usefulness is limited by the incomplete coverage of DT. To achieve unambiguous identification, the use of analytical standards remains the most reliable approach. In this case, pure VOC standards and a VOO sample were injected under the same experimental conditions. Then, the RI and DT of the standards’ features were directly compared with those of unknown sample features in order to evaluate the potential of the standards for VOC identification using GC-IMS.

First, the 45 VOC standards (previously described in the “Reagents and Standards” portion of the “Materials and Methods” section) were individually analyzed at 1 ppm in water using the GC-IMS instrument, in order to establish the RI and DT of their features. Subsequently, the same VOCs were injected together in a single vial at the same concentration. The plots obtained from the analysis of the mixture of 45 standards revealed a total of 84 features, whose identification was attempted using two complementary strategies: (a) using the library, or (b) through the comparison of their RI and DT with those of the standards that were analyzed individually. Then, we evaluated how many of the 45 standards in the mix could be putatively identified through the library alone, and which required additional confirmation with the analysis of their individual counterparts.

Table 5 summarizes which features were identified in the mixture of 45 VOC standards, and how that identification was achieved. In total, 48 features corresponding to 34 VOCs were identified in the mixture (putatively with the library and/or confirmed with standards), whereas the features of 11 VOCs were not. For 15 of the 34 identified VOCs, both the monomer and dimer were located, whereas for the remaining 19 identified VOCs, only one ion (either monomer or dimer) was observed. Unknown ions in Table 5 might correspond to fragments with higher mobility than the hydrated protons that constitute the RIP, but it was not confirmed.

**Table 5:** List of 34 Features That Were Identified in the Mixture of 45 Standards Using the Library or a Comparison of Features of Standards Analyzed Individually^a^

VOC feature	RI^a^	DT (RIP relative)	Method
**Alcohols**			
2-methyl-3-buten-2-ol (D)	631.5	1.216	Standards
2-methyl-3-buten-2-ol (U)	631.9	0.948	Standards
1-penten-3-ol (U)	682.2	0.942	Standards
*cis*-2-penten-1-ol (U)	774.3	0.942	Standards
pentan-1-ol (M)	779.9	1.251	Standards
3,3-dimethylbutan-1-ol (M)	799.6	1.308	Standards
hexan-1-ol (D)	869.6	1.634	Library
hexan-1-ol (M)	869.8	1.326	Library
1-octen-3-ol (D)	989.7	1.592	Library
octan-1-ol (M)	1083.9	1.465	Library
**Aldehydes**			
pentanal (D)	700.8	1.422	Library
pentanal (M)	700.8	1.185	Library
*trans*-penten-2-al (D)	752.5	1.361	Standards
*trans*-penten-2-al (M)	752.8	1.110	Standards
hexanal (D)	799.2	1.562	Library
hexanal (M)	799.5	1.259	Library
(E,E)-2,4-hexadienal (D)	908.3	1.449	Standards
benzaldehyde (D)	965.6	1.468	Library
nonanal (D)	1119.1	1.937	Library
**Ketones**			
diacetyl (D)	609.7	1.175	Library
butan-2-one (D)	622.0	1.246	Library
butan-2-one (M)	622.6	1.061	Library
pentan-2-one (D)	695.0	1.372	Library
pentan-2-one (M)	695.3	1.121	Library
4-methylpentan-2-one (M)	736.2	1.180	Standards
hexan-2-one (D)	792.0	1.503	Library
hexan-2-one (M)	792.2	1.192	Library
heptan-2-one (D)	889.0	1.632	Library
heptan-2-one (M)	889.3	1.261	Library
cyclohexanone (D)	889.3	1.454	Library
cyclohexanone (M)	889.8	1.156	Library
1-octen-3-one (M)	987.0	1.272	Standards
6-methyl-5-hepten-2-one (M)	997.4	1.175	Standards
octan-2-one (D)	1003.0	1.757	Library
nonan-2-one (D)	1097.0	1.875	Library
nonan-2-one (M)	1097.3	1.403	Library
**Esters**			
ethyl propanoate (D)	712.3	1.455	Library
methyl butanoate (D)	721.9	1.430	Standards
ethyl isovalerate (M)	852.5	1.257	Standards
ethyl isovalerate (D)	851.8	1.653	Standards
ethyl hexanoate (D)	1010.2	1.797	Standards
heptyl ethanoate (D)	1115.5	2.025	Standards
**Others**			
acetic acid (D)	647.3	1.145	Library
acetic acid (M)	647.7	1.057	Library
2-methylpyrazine (D)	824.6	1.386	Standards
2-methylpyrazine (M)	825.6	1.073	Standards
2,5-dimethylpyrazine (D)	910.9	1.491	Library
R-limonene (M)	1035.9	1.218	Standards

a Legend: (M), monomer; (D), dimer; (U), unknown ion before the RIP.

b Calculated using C4–C9 *n*-ketones.

The features of 9 of the 11 unidentified VOCs present in the mixture were affected by overlapping: heptan-2-ol, heptanal, propyl butyrate, 2-methylbutan-1-ol, 3-methylbutan-1-ol, octan-3-ol, octan-3-one, butanal, and 2-methylpropanal. Two types of overlapping were observed: (a) where the dimer feature overlapped and no monomer was observed (heptan-2-ol/heptanal/propyl butyrate, as well as 2-methylbutan-1-ol/3-methylbutan-1-ol), and (b) where the features of both ionic species overlapped (octan-3-ol/octan-3-one, and butanal/2-methylpropanal). Moreover, the features of octanal and ethyl acetate were not detected in the mixture despite the apparent absence of overlapping (based on the RT and DT values of their individual standards) probably due to competitive effects in ion generation with other VOCs within the mixture.

On the other hand, the features of 18 of the 34 located VOCs were identified using only the library. This was made possible mainly because the DT of their ions were available in the library. On the contrary, the remaining 16 of the 34 located VOCs required additional confirmation of their identity by analyzing their individual standards. Of these 16 VOCs, 11 required confirmation because the library lacked their DT values (3,3-dimethylbutan-1-ol, 1-octen-3-one, (E,E)-2,4-hexadienal, ethyl hexanoate, methyl butanoate, heptyl ethanoate, 1-penten-3-ol, 2-methyl-3-buten-2-ol, *cis*-2-penten-1-ol, *trans*-penten-2-al, and ethyl isovalerate). For the remaining 5 VOCs, DT values were available in the library, but confirmation with standards was necessary due to ambiguities in their identification. These consisted of feature overlapping or the proximity of features of multiple VOCs within the selected search margins. As an example, overlapping impeded the identification of pentan-1-ol, 4-methylpentan-2-one, and 6-methyl-5-hepten-2-one. In summary, the analysis of individual standards proved fundamental to detect overlapping features of different VOCs, preventing erroneous identifications.

As highlighted in a previous section, feature overlapping can be partially addressed by the generation of several ionic species from a single VOC. For instance, while 4-methylpentan-2-one, 2-methylbutan-1-ol and 3-methylbutan-1-ol shared similar values of RI (∼737–741) and dimer DT (∼1.46–1.49), they showed different monomer DT. However, when mixed together, the monomers of 2-methylbutan-1-ol and 3-methylbutan-1-ol were barely visible, as the ion equilibrium was displaced to the dimer generation. Therefore, while GC-IMS double separation is an advantage, it does not always solve overlapping issues.

### Dual Approach for VOCs Identification: Library and Standard Validation

As previously stated, the use of the library provides a valuable starting point for compound identification in samples. When both RI and DTs values are available, the library can greatly facilitate VOC assignment. However, when many compounds share similar RI values, and no discriminating parameter such as DT is present, annotation is uncertain. In such cases, validation using analytical standards is essential. In the present study, VOO was selected as a model sample to evaluate the complementarity of both strategies for VOC identification. Its volatile fraction is highly diverse, comprising aldehydes, ketones, alcohols, and esters, which makes VOO a challenging but informative system for testing targeted identification approaches.^9,22,73^ The standard mixture of 45 compounds, many of which have been reported in olive oil,^22^ was used for comparison and confirmation of detected VOCs.

Figure 2 shows a topographic plot of the VOO sample analyzed by GC-IMS. A total of 167 features were located in the sample, 45 of which were assigned to monomers and/or dimers of 34 VOCs (see Table S1) using the library and/or standards. Of these 45 features, 38 corresponding to 28 VOCs were putatively identified using the library with the previously recommended search margins of ±5 for RI and ±0.02 for DT (only if DT values were available). In addition, 29 of the 45 features, corresponding to 21 VOCs, were identified using analytical standards. Of these 29 features, 22 were already identified using the library, which confirmed their identity. Seven features (corresponding to 6 VOCs) of the 29 features that were identified with standard analysis did so exclusively, thanks to this method, as they could not be identified using the library. It was the case of those of, e.g., 1-penten-3-ol, *trans*-penten-2-al, methyl butanoate, 3,3-dimethylbutan-1-ol, 3-methylbutan-1-ol, and benzaldehyde. The identification of these VOCs required standard analysis because their DT values were not available in the library. Likewise, a single feature was associated by the library to benzaldehyde and 1-octen-3-ol monomers (with DT values available for both compounds). In this case, the individual analysis of both standards was required to confirm benzaldehyde as the correct identity, as shown in Figure S2. On the other hand, the identity of 16 features (corresponding to 13 VOCs) of the 22 features that were identified using the library could not be confirmed using standards because their corresponding standards were not included in this study.

**Figure 2:**
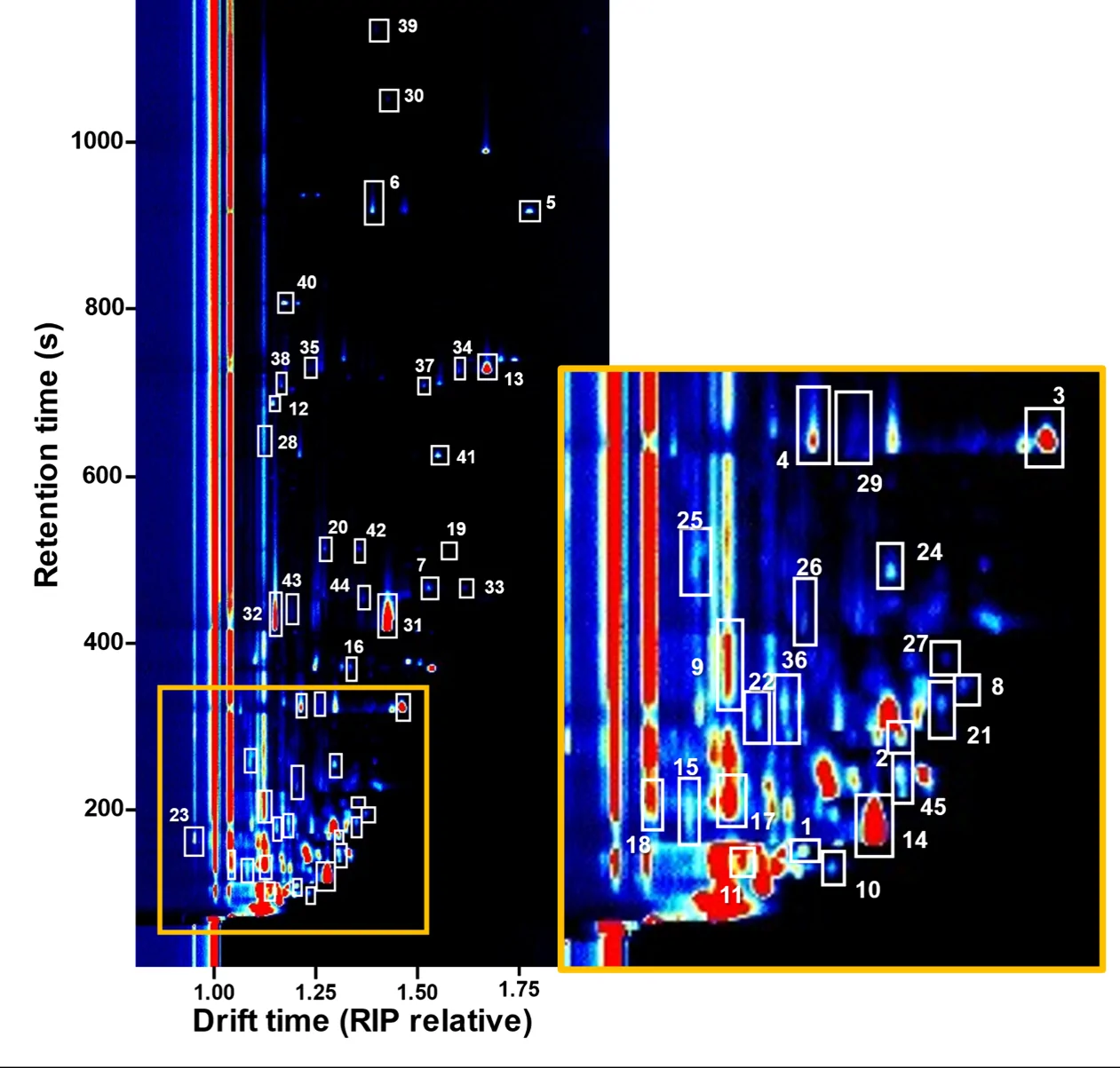
GC-IMS topographic plot of the VOO sample analyzed. A total of 167 features were located. Legend: 1, butan-2-one (D); 2, pentan-2-one (D); 3, hexanal (D); 4, hexanal (M); 5, nonanal (D); 6, nonanal (M); 7, hexan-1-ol (D); 8, ethyl propanoate (D); 9, ethyl propanoate (M); 10, butanal (D); 11, diacetyl (D); 12, 6-methyl-5-hepten-2-one (M); 13, octanal (D); 14, ethyl acetate (D); 15, ethyl acetate (M); 16, 2-methylpyrazine (D); 17, acetic acid (D); 18, acetic acid (M); 19, heptanal (D); 20, heptanal (M); 21, pentanal (D); 22, pentanal (M); 23, 1-penten-3-ol (before RIP); 24, *trans*-penten-2-al (D); 25, *trans*-penten-2-al (M); 26, 3-methylbutan-1-ol (M); 27, methyl butanoate (D); 28, benzaldehyde (M); 29, 3,3-dimethylbutan-1-ol (M); 30, nonan-1-ol (M); 31, 2-hexen-1-ol (D); 32, 2-hexen-1-ol (M); 33, 3-methylbutyl acetate (D); 34, 1,8-cineole (D); 35, 1,8-cineole (M); 36, 2,3-pentanedione (M); 37, β-pinene (D); 38, β-pinene (M); 39, decan-2-one (M); 40, 2,5-dimethyl-4-hydroxy-3­[2H]-furanone (M); 41, hexanoic acid (D); 42, ethenyl benzene (D); 43, *cis*-3-hexen-1-ol (M), 44: styrene (M), 45: butan-1-ol (D).

A particularly illustrative case of the importance of validating with analytical standards is 3-methylbutan-1-ol (Figure 3). For the actual feature of its dimer, identified with the standard, the library originally suggested two additional candidates: the dimers of 2-methylbutan-1-ol and 4-methylpentan-2-one. However, as the library did not include DT values for 3-methylbutan-1-ol (monomer or dimer), this compound was not retrieved as a candidate. Nevertheless, comparison with its individual standard confirmed the presence of the 3-methylbutan-1-ol monomer.

**Figure 3:**
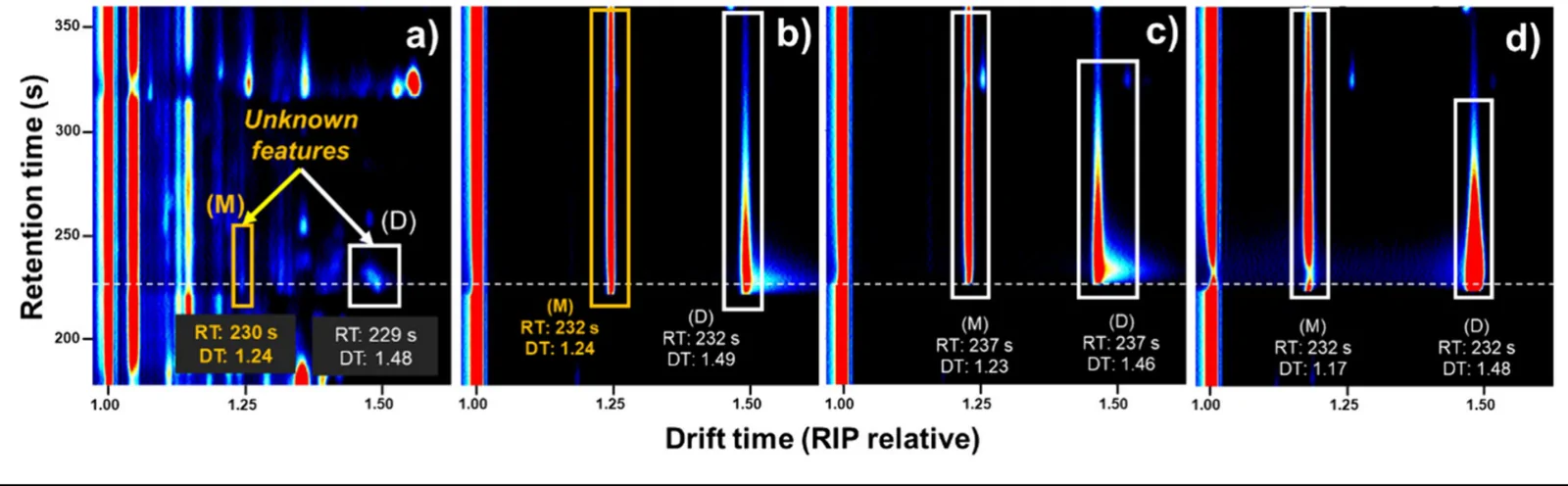
GC-IMS topographic plots (a) VOO sample, (b) 3-methylbutan-1-ol, (c) 2-methylbutan-1-ol, and (d) 4-methylpentan-2-one.

In summary, although 34 VOCs were identified in the VOO sample using the library and/or the analysis of standards, only the identity of 21 of these could be validated with certainty through a dual approach consisting on a library search and subsequent analysis of standards. Full confirmation of VOC identities would ultimately require either the injection of all analytical standards retrieved by the library and the use of more powerful instrumentation, such as high-resolution mass spectrometry. However, these strategies are both labor-intensive and costly, which limits their application in routine laboratories. Despite these considerations, in practice, a considerable number of studies rely solely on commercial libraries for identification (see Table 1), which keeps confidence in GC-IMS identification at level 2 at most (annotation).

Therefore, for identification to be feasible it is crucial to specify whether RIs alone or RIs and DTs were used, the search margins applied in the library and confirm the identification using standards. The number of features identified in complex samples and the reliability of this identification depends strongly on the chosen strategy. In the present study, the combined use of the library and analytical standards resulted in the putative identification of 45 features (26.9% of the 167 detected). Of these 45 identified features, only 29 were confirmed after the analysis of 45 standards, yielding a final validated identification rate of 17.4%. By contrast, other studies (included in Table 1) that relied exclusively on library matches have reported considerably higher rates of identified features, such as 92.06% (58 out of 63 features).^69^ While others have successfully employed analytical standards for feature identification in GC-IMS workflows: As an example, in 2022, Li et al. analyzed standards using GC-MS, and the resulting assignments were then transferred to GC-IMS when retention times coincided.^45^ Similarly, direct comparison of the RI and DT values of samples’ features with those of standards proved to be an effective strategy, without the need to rely on the library at all.^25^

The combined use of library-based annotation and subsequent confirmation with analytical standards proposed in this study is not without precedent in the GC-IMS literature, although a systematic evaluation of its implementation has been lacking. Several research groups have employed hybrid strategies in which a library search provided an initial set of candidates that are subsequently validated through independent injection of reference compounds. As examples, standard-based assignments have been also transferred from GC–MS to GC-IMS by correlating retention times, effectively using the first technique to extend the confirmed identification list of the second.^39^ In addition, in 2025, Navarro-Laguna et al. compared HS-GC-IMS and HS-SPME-GC-MS for microbial VOC identification, using standard injection for Level 1 confirmation in both platforms demonstrating the complementarity of both techniques.^13^ Notably, the GC-IMS library search in that study was performed including search margins as recommended in the present work, and all annotated compounds were confirmed with their corresponding analytical standards, an approach aligned with the dual workflow proposed here. Similarly, combined HS-GC-IMS and HS-GC-MS workflows in multiple food matrices have confirmed GC-IMS identifications with standards.^14^

In addition, in 2024, Weller and Parastar argued that GC-IMS is rapidly becoming a standard tool in volatile metabolomics workflows, but they noted that the absence of a formal identification framework analogous to those established in MS remains a gap limiting the comparability of published results.^74^ The framework proposed in the present study (Table 2) directly addresses this gap by adapting the confidence levels originally proposed for HRMS to the capabilities of GC-IMS.

### Recommended Workflow for GC-IMS Identification

Based on the experimental findings presented in this study, a structured framework for compound identification in GC-IMS is proposed to improve reliability and harmonize practices across laboratories.

*Step 1: Define identification confidence levels before starting.* A clear distinction must be established between putative annotation and confirmed identification (see Table 2). Consistent with established practices in mass spectrometry, library-based matches using RI and DT should be considered as Level 2 (putative annotation), whereas only the comparison with analytical standards under identical instrumental conditions can provide Level 1 (confirmed identification). This distinction should be reported explicitly in any publication.

*Step 2: Normalize RIs.* Prior to any library query, raw RTs must be converted to RIs using a homologous series of reference compounds (*n*-ketones, when a nonpolar GC column is used) analyzed under identical conditions. This step is essential to reduce interlaboratory and interinstrument variability and is required regardless of the software platform used.

*Step 3: Perform library screening with combined RI and DT.* Query the available database by defining a search window centered on the experimental RI and DT of the unknown feature. Based on the results of the present study, a tolerance of ±5 RI units and ±0.02 RIP-relative DT is recommended as a starting point. If DT values are not available in the database, the RI-only search should be treated as low-confidence screening rather than putative identification. The presence of multiple ionic species of the same VOC may provide complementary evidence and should be used to support (or challenge) library candidates.

*Step 4: Confirm with analytical standards.* Library candidates should be confirmed by injecting the corresponding analytical standards under identical instrumental conditions and comparing their experimental RI and DT values with those of the unknown feature. This step is critical in cases of (a) overlapping features, (b) missing DT values in the database, or (c) multiple candidates within the search window. This is the only way of achieving Level 1 identification and of detecting competitive ionization artifacts or feature coelution that may lead to erroneous library assignments.

*Step 5: Report search parameters and identification confidence levels.* All published identifications should specify the search margins used for RI and DT, whether DT values were available in the library, whether analytical standards were injected, and the resulting confidence level according to the framework in Table 2. This transparency is essential for reproducibility and for meaningful cross-study comparisons. Based on these considerations, three levels of confidence for GC-IMS annotation can be defined in practice: (i) preliminary screening based on RI only (low confidence), (ii) putative annotation based on combined RI and DT matching (moderate confidence), and (iii) confirmed identification through comparison with analytical standards (high confidence).

For applications requiring further confirmation, the integration of complementary analytical techniques such as GC-MS should be considered, as they provide orthogonal molecular information that substantially reduces identification ambiguity. A schematic overview of this workflow is presented in Figure S3.

## Conclusions

This study demonstrates the potential and the limitations of GC-IMS for the identification of VOCs features in complex matrices, addressing gaps in current literature regarding signal assignment on topographic plots. Our findings demonstrate that putative identification requires a multiparametric approach; relying exclusively on RI values is insufficient. Instead, DT values must be also in whenever available, applying precise search margins. This work proposes optimized search thresholds of ±0.02 for DT and ±5 for RI, to maximize identification accuracy, but these values should be revised when other methods are used.

A key contribution of this study is the experimental validation of a dual annotation strategy, showing that the combined use of library matching and analytical standards is necessary to achieve robust and unambiguous compound identification in complex matrices. In addition, this work introduces a structured framework for confidence levels in GC-IMS, inspired by mass spectrometry, which clarifies the distinction between putative annotation and confirmed identification. This contributes to a more consistent and harmonized interpretation of GC-IMS results across studies.

Beyond analytical considerations, we have detected a lack of transparency in published studies, where the absence of reported search parameters has compromised reproducibility, particularly when only a library was used for annotation. While the formation of monomers and dimers in GC-IMS enhances discrimination, our analysis confirms that overlapping signals and ion competition prevail. Consequently, library matching must be regarded just as a preliminary screening tool that requires further validation through analytical standards. This is crucial to reveal hidden interferences that may be overlooked by library-based approaches.

The application of this dual strategy to a VOO sample confirmed the inherent limitations of VOC identification with GC-IMS when the number of available analytical standards is finite. Out of 167 detected features detected in the test VOO sample, only 29, corresponding to 21 VOCs, were confirmed by both approaches, yielding a validated identification rate of approximately 17% under the experimental conditions of this study. This figure should not be interpreted as a universal performance ceiling for GC-IMS identification; rather, it reflects the coverage achievable when 45 standards, selected based on known constituents of olive oil and related matrices, are used to validate library candidates. Laboratories working with more comprehensive standard sets, or focusing on a narrower set of target analytes, may achieve substantially higher identification rates. Nevertheless, the result stands in contrast to the identification rates claimed in the recent (2015–2026) most-cited (>100) literature, where the reliance on library-only annotation without standard confirmation has led to reported rates of feature identification of up to 92%, a figure that this study suggests corresponds, at best, to Level 2 (putative annotation) rather than confirmed identification.

Ultimately, this work highlights that while enhanced libraries are vital, they cannot yet replace manual intervention. The adoption of a standardized protocol and the systematic use of analytical standards are imperative to transition GC-IMS to a confirmatory analytical method for complex samples. Future efforts should focus on the expansion of open-access DT databases and the implementation of more advanced deconvolution algorithms for GC-IMS features.

## Supplementary Material



## Notes

Table S1: List of 45 features corresponding to 34 VOCs identified in the VOO sample through the use of the library, standards, or both strategies. Figure S1: GC-IMS plots of 2-methyl-3-buten-2-ol, 1-penten-3-ol, and *cis*-2-penten-1-ol, at 1 mg/L in ultrapure water. Figure S2: GC-IMS topographic plots of VOO sample, benzaldehyde, and 1-octen-3-ol. Figure S3: Recommended workflow for feature identification in GC-IMS (PDF)

## Abbreviations

GC: Gas chromatography
IMS: Ion mobility spectrometry
VOCs: Volatile organic compounds
DT: Drift time
RI: Retention index
RIP: Reactant ion peak
MS: Mass spectrometry
VOO: Virgin olive oil
HRMS: High-resolution mass spectrometry
PA: Proton affinity
HS: Headspace
CCS: Collision cross-section
