# Supplementary material for: Analytical Challenges and Strategies for Compound Identification Using Gas Chromatography–Ion Mobility Spectrometry
Source: Anal Chem. 2026 Aug 3;98(32):23426–39. doi: 10.1021/acs.analchem.5c07242 (PMC13492047; doi:10.1021/acs.analchem.5c07242)
Supplement: Supplementary file 1 [file ac5c07242_si_001.pdf]

Analytical challenges and strategies for compound identification using gas chromatography-ion mobility spectrometry

Andrés Martín-Gómez, María José Cardador, Laura Pérez-González, and Lourdes Arce*

Department of Analytical Chemistry, Chemical Institute for Energy and the Environment (IQUEMA), University of Córdoba, Campus of Rabanales, Marie Curie Annex Building, E-14071, Córdoba, Spain

*Corresponding author: qa1arjil@uco.es

Table of contents

Table S1. List of 45 features corresponding to 34 VOCs identified in the VOO sample through the use of the library, standards or both strategies. M: Monomer, D: Dimer.

Figure S1. GC-IMS plots of a) 2-methyl-3-buten-2-ol, b) 1-penten-3-ol, and c) cis-2-penten-1-ol, at 1 mg/L in ultrapure water.

Figure S2. GC-IMS topographic plots a) VOO sample, b) benzaldehyde, and c) 1-octen-3-ol.

Figure S3. Recommended workflow for feature identification in GC-IMS.

Table S1. List of 45 features corresponding to 34 VOCs identified in the VOO sample through the use of the library, standards or both strategies. M: Monomer, D: Dimer.

Nº	Feature	RI*	DT (RIP relative)	Method**
45	1-butanol (D)	667.0	1.368	Library
36	2,3-pentanedione (M)	701.4	1.222	Library
31	2-hexen-1-ol (D)	854.2	1.517	Library
43	(Z)-3-hexen-1-ol (M)	857.0	1.228	Library
32	2-hexen-1-ol (M)	854.5	1.182	Library
44	styrene (M)	866.1	1.445	Library
33	3-methylbutyl acetate (D)	873.6	1.746	Library
42	ethenyl benzene (D)	897.7	1.431	Library
41	hexanoic acid (D)	961.9	1.668	Library
38	beta-pinene (M)	1008.6	1.202	Library
37	beta-pinene (D)	1008.7	1.623	Library
35	1,8-cineol (M)	1017.7	1.283	Library
34	1,8-cineol (D)	1018.9	1.726	Library
40	2,5-dimethyl-4-hydroxy-3[2h]-furanone (M)	1054.8	1.206	Library
30	1-nonanol (M)	1171.7	1.522	Library
39	2-decanone (M)	1210.5	1.481	Library
10	butanal (D)	606.0	1.280	Library + Standards
11	diacetyl (D)	610.2	1.166	Library + Standards
1	2-butanone (D)	622.0	1.246	Library + Standards
15	ethyl acetate (M)	638.1	1.097	Library + Standards
14	ethyl acetate (D)	638.7	1.338	Library + Standards
17	acetic acid (D)	649.0	1.148	Library + Standards
18	acetic acid (M)	650.6	1.053	Library + Standards
2	2-pentanone (D)	695.0	1.371	Library + Standards
21	n-pentanal (D)	700.8	1.422	Library + Standards
22	n-pentanal (M)	700.8	1.185	Library + Standards
8	ethyl propanoate (D)	711.1	1.452	Library + Standards
9	ethyl propanoate (M)	710.9	1.150	Library + Standards
3	hexanal (D)	797.0	1.560	Library + Standards
4	hexanal (M)	797.0	1.256	Library + Standards
16	2-methylpyrazine (D)	822.9	1.406	Library + Standards
7	1-hexanol (D)	873.6	1.637	Library + Standards
19	heptanal (D)	899.1	1.692	Library + Standards
20	heptanal (M)	898.6	1.327	Library + Standards
12	6-methyl, 5 hepten-2-one (M)	997.0	1.173	Library + Standards
13	octanal (D)	1018.3	1.811	Library + Standards
5	1-nonanal (D)	1107.0	1.939	Library + Standards
6	1-nonanal (M)	1108.2	1.470	Library + Standards
23	1-penten-3-ol (before RIP)	682.2	0.942	Standards
27	methyl butanoate (D)	721.9	1.430	Standards
26	3-methyl-1-butanol (M)	735.7	1.246	Standards
24	(E)-2-pentenal (D)	752.5	1.361	Standards
25	(E)-2-pentenal (M)	752.8	1.110	Standards
29	3,3-dimethyl-1-butanol (M)	799.6	1.308	Standards
28	benzaldehyde (M)	965.9	1.152	Standards

*Calculated using C4-C9 n-ketones, **Identification method employed

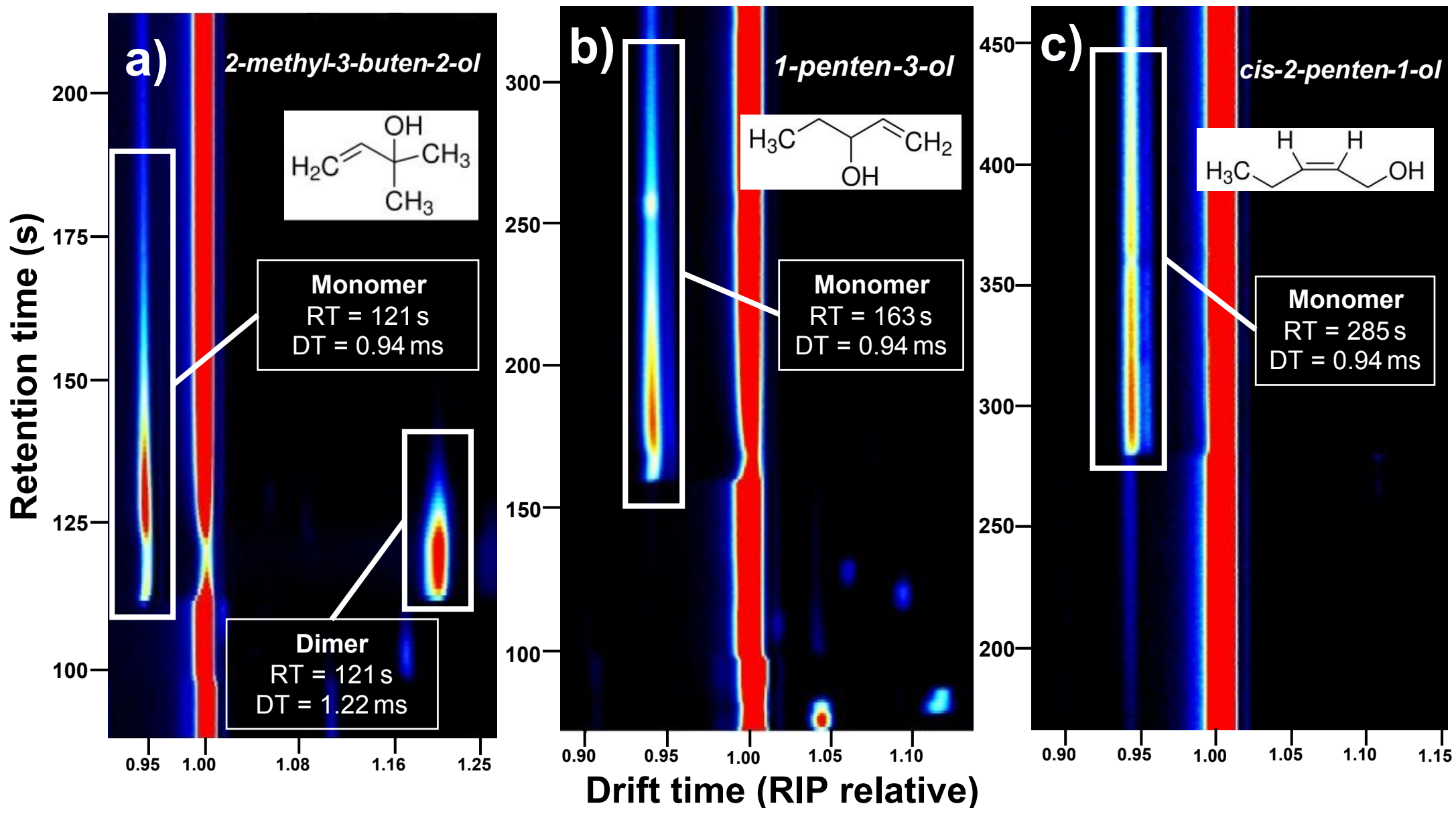

Figure S1. GC-IMS plots of a) 2-methyl-3-buten-2-ol, b) 1-penten-3-ol, and c) cis-2-penten-1-ol, at 1 mg/L in ultrapure water.

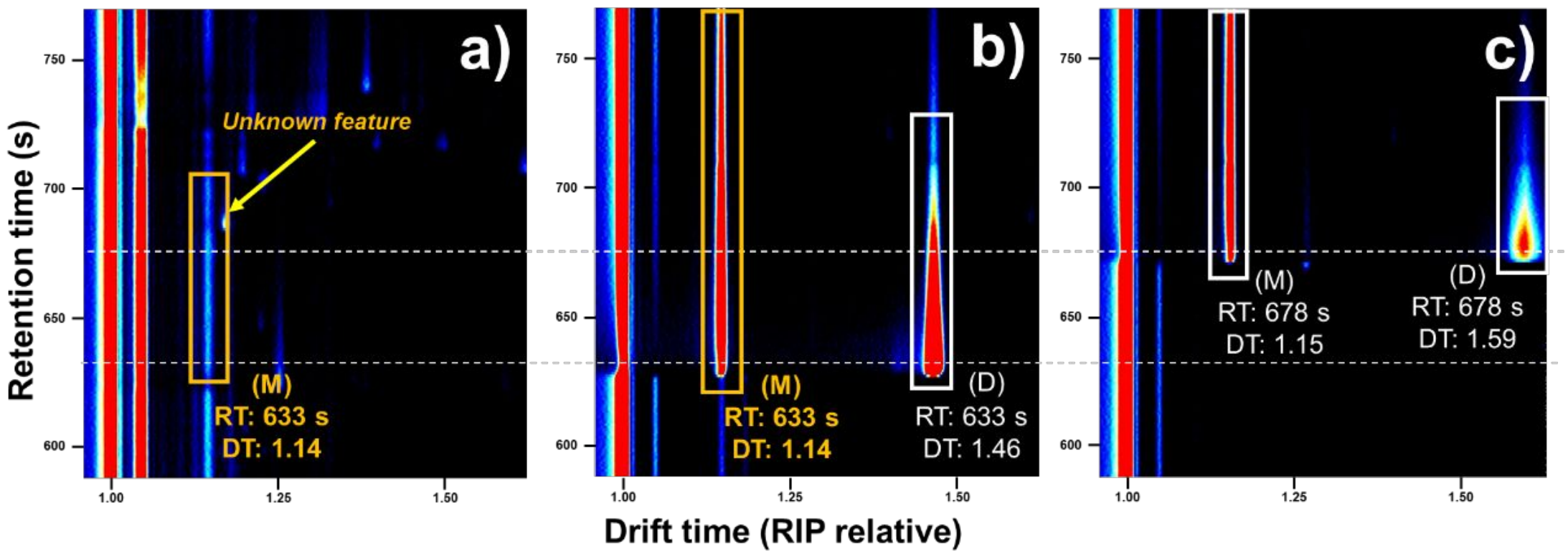

Figure S2. GC-IMS topographic plots a) VOO sample, b) benzaldehyde, and c) 1-octen-3-ol.

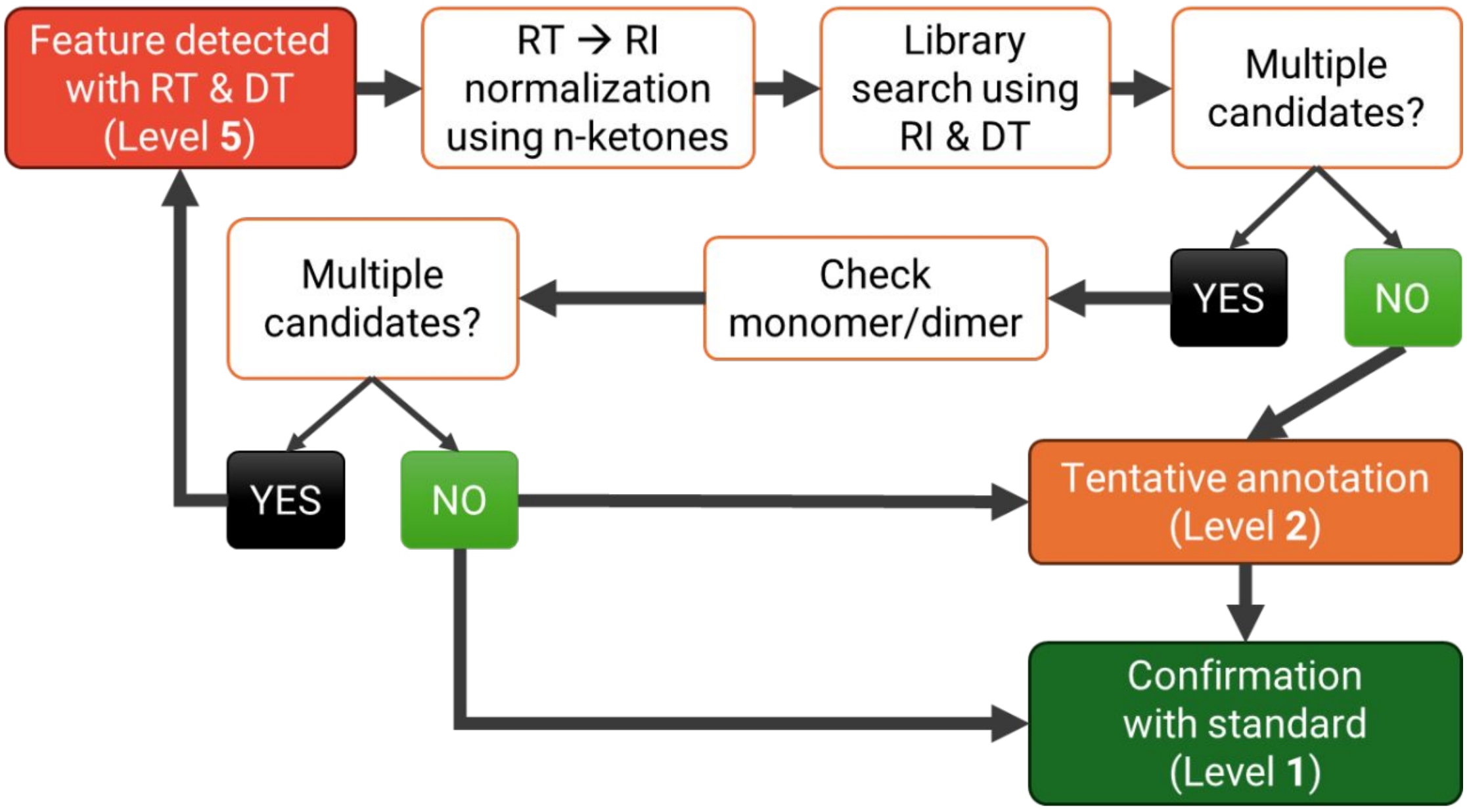

Figure S3. Recommended workflow for feature identification in GC-IMS.
